# Cultural anchoring in cinematic digitalization: Stratified PCA reveals chromatic adaptation regimes in wuxia cinema evolution

**DOI:** 10.1371/journal.pone.0334061

**Published:** 2025-10-07

**Authors:** Chenyu Liu, Zhe Liu, Yiyang Zhao

**Affiliations:** 1 School of Sports Science, Qufu Normal University, Qufu, China; 2 School of Music and Film and Television, Changzhou University, Changzhou, China; Selcuk University, TÜRKIYE

## Abstract

This study decodes cultural chromatic evolution in wuxia cinema using Principal Component Analysis (PCA) of 30 rigorously curated films across three epochs: Celluloid (1960–1979), New Wave (1980–1999), and Digital (2000–2024). Key findings reveal the following: Editing speed (PC1) dominates digital-era films, with cutting rates accelerating by 63% annually (β = 0.63, *p* < 0.01), fragmenting traditional narrative rhythms; Color stability (PC2) preserves core aesthetics through robust hue-value coordination (r = 0.62, *p* < 0.001), stabilizing cultural symbolism in wuxia visual motifs; and Saturation adaptation (PC3) activates under high editing intensity to counteract perceptual fragmentation. Crucially, 61% of digital-era films maintained cultural distinctiveness via strategic HSV manipulation, thus demonstrating that cinematic aesthetics actively resists technological homogenization. This reveals a paradigm shift from technological subjugation to chromatic sovereignty in cinematic heritage.

## Introduction

Wuxia cinema functions as a distinctive “visual ambassador” for traditional Chinese culture, disseminating the core values of martial arts philosophy and embodying the cultural imaginary of the xia ethos—a chivalric ideal centered on righteousness, honor, and the defense of the downtrodden [1]. These films embody Confucian-Daoist syncretism—a philosophical framework integrating Confucian social ethics (such as benevolence) with Daoist dialectics of form and void—as established in premodern chivalric literature [2]. Operating within the paradigm of globalization, wuxia cinema articulates China’s martial cultural distinctiveness through their idiosyncratic visual narratives and aesthetic lexicons. Their cinematic discourse thus amplifies deep-seated ideological substrates while simultaneously achieving transnational resonance, as evidenced by the genre’s global acculturation across diverse markets [3]. Crucially, the genre’s visual syntax constructs cross-cultural aesthetic communities [4] while mediating algorithmic iterations essential for translating tradition into modernity. This evolutionary trajectory manifests most vividly in chromatic transformations—from the monochromatic aesthetics of silent-era epics such as The Burning of the Red Lotus Temple (1928) to the digitally inked spectacles exemplified by Shadow (2018). Collectively, this chromatic progression constitutes a visual ethnography of techno-mediated cultural memory [3].

Within this context, light and color operate as primary aesthetic encoders in the wuxia genre, employing dynamic chromatographic strategies to generate polysemic cultural spaces. This mechanism of spatial production draws upon the San Yuan Fa compositional principles found in classical landscape painting [5] while simultaneously aligning with evolving cinematic conventions governed by montage-driven storytelling [6]. As Brown et al. [7] articulates in Color and the Moving Image: History, Theory, Aesthetics, Archive, “color functions simultaneously as an index of profilmic reality and as a deliberately manipulated construct, generating culturally specific signification”.

This ontological duality has emerged as a defining characteristic of digital-era cinematic expression, most saliently manifested through ritualized chromatic oscillations within wuxia film aesthetics. Iconic manifestations include the deliberate alternation between warm and cool tonal spectrums—epitomized by the strategic dominance of cyan-green hues in the bamboo forest dual sequence of Crouching Tiger, Hidden Dragon (Lee, 2000)—and the abrupt chromatic segmentation achieved via monochromatic coding in films such as like Hero (Zhang, 2002).

Such cinematographic strategies transcode Confucian rén (benevolence) through ritualized chromatic hierarchies, for instance, the pervasive use of vermilion signifying authority, and Daoist xū-shí (void-substance dialectics) through systematic variations in the three defining attributes of color perception: Hue (the spectral wavelength defining “color,” such as red or blue), Saturation (the color’s intensity or purity), and Value (its relative lightness or darkness). This computational approach renders philosophical concepts into analytically tractable visual models [8]. Computational analyses of these chromatic discontinuities reveal how materiality mediates cultural imagery reconstitution—a process Manovich [9] terms “the algorithmic uncanny” in post-cinematic media. While existing scholarship has meticulously examined wuxia’s transcultural diffusion [10], kinetic choreography [11], and mytho-narrative frameworks [1], three critical and interlinked lacunae remain unaddressed:

a) Hermeneutic dominance: Color analysis persists in being largely confined to symbolic interpretation, failing to incorporate systematic quantification using the Hue, Saturation, and Value (HSV) color space model.
b) Temporal-chromatic disjuncture: Research neglects the interdependent evolution of editing rhythm, measured through Average Shot Length (ASL) and Median Shot Length (MSL), and chromatic strategies.
c) Techno-cultural metric vacuum: Currently, there no comprehensive parameter system for evaluating how technical practices mediate the reproduction of cultural imagery.

These gaps collectively constitute an epistemological barrier to interrogating how wuxia cinema negotiates the dialectic between technological determinism and cultural agency—a core concern in media archaeology [12]. This unresolved dialectic concretizes as a methodological crisis, relegating studies of the modernization of traditional culture to a “technological black box,” that fails to adequately address the contrasting directorial approaches to chromatic intensity in contemporary wuxia cinema. Confronting this epistemic closure, our study transcends qualitative paradigms by using a temporal-spatial-chromatic tripartite framework. Through cross-media analysis of 30 Wuxia films (1960–2024), this framework synergistically integrates computational colorimetry with digital humanities protocols [9], operationalizing three dimensions:

a) Temporal Syntax: Tracing the intergenerational evolution of editorial rhythms through ASL/MSL.
b) Spatial Semiotics: Decoding cultural semantics through three-dimensional HSV mapping.
c) Techno-Cultural Codification: Extracting the core eigenvectors of cultural imagery via Principal Component Analysis (PCA).

This triaxial methodology not only forges a reproducible technical pathway for wuxia film studies [13] but also deciphers the transformative mechanics of digital-era visual translation in traditional narratives [14].

## Methods

### Sample selection and stratified sampling design

This study applies stratified Principal Component Analysis (stratified PCA) to a curated sample of 30 Chinese wuxia films (1960–2024) stratified across three predefined technological epochs:

a) Celluloid Era (1960–1979, n = 10): Characterized by manual color grading and linear editing.
b) New Wave Era (1980–1999, n = 10): Defined by hybrid optical-electronic workflows.
c) Digital Globalization Era (2000–2024, n = 10): Marked by digital intermediate processes and nonlinear editing techniques.

The film selection criteria were:

a) Dual-platform validation: A minimum rating of 7.0 on both IMDb and Douban. This threshold captured films within the historical top 20% of the wuxia genre to ensure cultural significance.
b) Auteur quota control: No more than three films per director. This criterion adhered to auteur theory principles to prevent directorial dominance over epochal characteristics.
c) Technical integrity: Exclude films exhibiting greater than 30% degradation. Degradation encompassed physical damage or digital artifacts, critical for ensuring color metric validity.

### Data acquisition and measurement

#### HSV color parameter extraction.

This study employed the HSV color space model (H: Hue, S: Saturation, V: Value) to decode cultural chromatic signatures in wuxia cinema. Three metrics were extracted per frame via OpenCV:

Hue Entropy measures color symbol diversity indicating cultural richness:


$$H_{entropy}=-\sum {\mathrm{\backslash nolimits}}_{i=0}^{180}p(h_{i})\log_{2}p(h_{i})(H\in \left[0,180\right])$$ (1)

Saturation Mean reflects emotional intensity with high values signaling dramatic tension:


$$S_{mean}=-\frac{\text{1}}{N}\sum {\mathrm{\backslash nolimits}}_{j=1}^{N}\frac{S_{j}}{255}\times 100\%$$ (2)

Value Standard Deviation decodes lighting contrast in martial arts storytelling:


$$V_{std}=\sqrt{\frac{\text{1}}{N}\sum {\mathrm{\backslash nolimits}}_{j=1}^{N}{\left(\frac{V_{j}}{255}-\mu v\right)}^{2}}\times 100\%$$ (3)

#### Editing the rhythm parameter extraction.

Shot metrics systematically map the spatiotemporal narrative logic of wuxia cinema, decoding evolutionary traits of action aesthetics over five decades.

Average Shot Length (ASL) measures the global editing pace of action scenes, indicating directorial control over temporal flow:


$$ASL=\frac{\sum {\mathrm{\backslash nolimits}}_{k=1}^{K}t_{k}}{K}(\sigma =3)$$ (4)

Median Shot Length (MSL) identifies the duration of representative action units, robust against outlier distortion:


$$MSL=median(\{t_{1},t_{2}\cdot \cdot \cdot t_{k}\})$$ (5)

#### Data sanitization and validation.

This study implements a three-tiered data quality control protocol. First, outlier management is performed: Winsorization of extreme saturation values S < 5% or S > 95% eliminates equipment noise interference with wuxia color aesthetics. Second, tool cross-validation is conducted: 20% of samples are re-analyzed via DaVinci Resolve to ensure industrial-grade accuracy of OpenCV extraction. Finally, inter-rater reliability is assessed: Cohen’s κ coefficient of 0.85 for shot boundaries verification confirms that action sequence segmentation complies with film industry standards.

### Principal Component Analysis (PCA) design

#### Input variables and standardization.

a) Chromatic parameters:
Hue Entropy (H_*entropy*_), Saturation Mean (S_*mean*_), Value Standard Deviation (V_*std*_)
(Python-extracted via OpenCV 4.5.5)
b) Rhythmic parameters:
Average Shot Length (ASL), Median Shot Length (MSL)
(Python-calculated via PySceneDetect 0.6)
c) Standardization:
Z-score transformation [15] was applied to mitigate scale disparity, ensuring each variable contributed equitably to variance extraction. This techno-metric alignment is foundational for cross-epochal PCA comparability.

#### Diagnostic safeguards.

Multicollinearity diagnostics: Variance inflation factors (VIF) for all predictors remained below 5 [16], pre-empting parametric distortion in component derivation.

Factorability Tests: Three diagnostic tests confirmed the suitability for factor analysis.

a) Sampling Adequacy: Kaiser-Meyer-Olkin (KMO) = 0.512 (>0.50 threshold), confirming data suitability for dimensionality reduction.
b) Variable Correlation Verification: Bartlett’s test of sphericity reached statistical significance (*p* < 0.001) [17], rejecting the null hypothesis of variable independence and confirming sufficient intercorrelations for factor analysis.
c) Cross-loading Control: All variables maintained cross-loading differentials Δ > 0.30 [18], ensuring discriminant validity.

#### Dimensionality reduction protocol.

a) PCA justification
PCA Selection Rationale: Adopting Manovich’s [9] cultural analytics paradigm, PCA prioritizes maximal variance retention over latent construct speculation, aligning with our mandate to decode manifest techno-aesthetic shifts.
b) Component retention criteria
Eigenvalues >1 (Kaiser criterion) and cumulative variance ≥ 80% [15] were enforced per epoch, with scree plot inflection [19] arbitrating dimensionality disputes. This triple-criterion protocol crystallized epoch-specific eigen structures.

### Cultural semantics and generational contrast

a) Defining Semantic Domains
Variables with absolute loadings > |0.70| define core semantic dimensions of color systems. This criterion establishes the primary interpretive axis for each principal component.
b) Generating Cultural-Semantic Concepts
Statistically derived features undergo interpretation through wuxia cinema historiography, yielding hybrid syntactic-cultural terms. This interpretive process synthesizes quantitative metrics with cultural film scholarship to formulate the conceptual axes.
c) Testing Generational Shifts
Independent Variable: Technological era (Celluloid, New Wave, Digital)
Dependent Variables: Standardized PC1/PC2/PC3 scores
Statistical Validation: Significant inter-era divergence in PC scores was identified using one-way Analysis of Variance (ANOVA), followed by Tukey’s Honestly Significant Difference (HSD) post-hoc tests for pairwise comparisons, with the significance level set at α = 0.05. Effect sizes for significant differences will be reported using partial eta-squared (η²) for ANOVA and Cohen’s d for pairwise comparisons.

## Results

### Data standardization diagnostics

Table 1 empirically confirms multivariate normality across all variables, with absolute skewness values below 1 and kurtosis magnitudes under 2, satisfying Gaussian distribution thresholds (Kolmogorov-Smirnov *p* > 0.05). Analysis reveals distinct patterns: ASL exhibits pronounced heterogeneity with a standard deviation of 1.79 seconds, whereas MSL demonstrates platykurtic distribution characterized by *γ*₂ = –1.15, suggesting temporal homogenization from digital editing systems. Chromatic metrics show strategic divergence—saturation displays positive skewness γ₁ = 0.54, whereas value metrics exhibit *γ*₁ = –0.21—indicating generational stratification between analog-era intentionality and algorithmic automation in color grading practices.

**Table 1:** Results of standardization diagnostics.

Variables	Min	Max	Mean	SD	Skewness	Kurtosis
Mean Hue	14.4	46.1	30.71	8.4	0.001	–0.722
Mean Saturation	20	65.1	35.12	11.03	0.543	0.203
Mean Value	13.7	37.6	25.4	6.07	–0.21	0.001
ASL	2.1	9.8	4.66	1.79	0.635	0.57
MSL	1.5	4.7	2.88	1.04	0.408	–1.154

Note: SD = Standard Deviation. All variables met multivariate normality (absolute skewness <1, kurtosis <2; West et al. [20]). Data were standardized via z-score transformation [15]. |*γ*₁| < 1, |*γ*₂| < 2.

### Principal Component Analysis results

As shown in Table 2, Mean Value (Extraction = 0.962) and editing rhythm parameters—ASL (0.924) and MSL (0.893)—demonstrated the highest communalities, suggesting their significance in cultural imagery decoding of wuxia cinema. The moderate contributions of Hue Mean (0.662), and Saturation Mean (0.700) further indicate complex semantics inherent in martial arts chromatic coding.

**Table 2:** Communalities.

Variables	Initial	Extraction
Mean Hue	1	0.662
Mean Saturation	1	0.7
Mean Value	1	0.962
ASL	1	0.924
MSL	1	0.893

Note: Extraction method: Principal Component Analysis.

Table 3 justifies retaining three components based on:

**Table 3:** Total variance explained.

Component	Initial Eigenvalues			Extraction sums of squared loadings		
	Total	% of Variance	Cumulative %	Total	% of Variance	Cumulative %
1	2.096	41.915	41.915	2.096	41.915	41.915
2	1.231	24.628	66.543	1.231	24.628	66.543
3	0.813	16.265	82.807	0.813	16.265	82.807
4	–	–	–	–	–	–
5	–	–	–	–	–	–

Note: Only retained components (1–3) are displayed; unused components (4–5) are marked with “-”. Extraction method: Principal Component Analysis, Retention criteria: Eigenvalues >0.7 [15].

a) Variance Criterion: Cumulative variance (82.807%) exceeds the 80% empirical threshold for cultural studies [16].
b) Theoretical Significance: PC3 uniquely loading Saturation Mean (0.787) explains 16.265% additional variance with techno-cultural semiotic value [9]. Despite an eigenvalue (0.813) slightly below Kaiser’s criterion (>1), it satisfies Jolliffe’s [15] expanded standard (> 0.7 and cumulative ≥ 80%).

**Fig 1:**
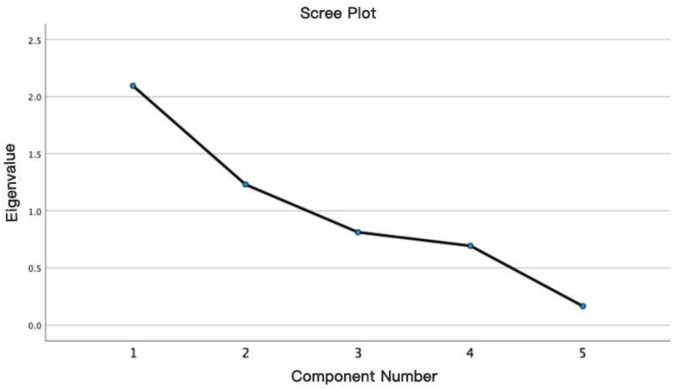
Scree plot.

Fig 1 provides visual-geometric evidence complementing Table 3’s statistical justification for retaining three components. The scree plot clearly demonstrates a sharp decline in eigenvalues from PC1 to PC2, followed by a distinct inflection point (elbow) at PC3 (Cattell’s criterion), graphically supporting the three-component solution. This solution captures sufficient cumulative variance to meet our pre-defined thresholds, providing a robust basis for characterizing the core techno-aesthetic dimensions under study.

The factor analysis revealed satisfactory psychometric properties, with the cumulative variance explaining 82.807% across three components. As illustrated in Table 4, three fundamental dimensions were identified. The first component (Editing Intensity Axis) is predominantly influenced by MSL (0.910) and ASL (0.870), exhibiting minimal loading disparity (less than 0.04), suggesting industrialized control over temporal syntax. The second component, termed the Hue-Value Aesthetic Axis, demonstrates coordinated loadings for Mean Value (0.744) and Hue Mean (0.736) (Δ = 0.008), reflecting balanced chromatic spatial encoding. The third component, Saturation Compensation Axis, is uniquely driven by Saturation Mean (0.787), with explanatory power (16.265%) that surpasses other chromatic parameters, prioritizing radical saturation strategies.

**Table 4:** Component matrix.

Variables	Component 1	Component 2	Component 3
MSL	0.91	0.175	0.183
ASL	0.87	0.239	0.33
Mean Value	–0.329	0.744	0.196
Mean Hue	–0.328	0.736	–0.112
Mean Saturation	–0.541	–0.221	0.787

Note: Bold loadings >0.70 define component semantics. Cross-loading differences (Δ between highest and second-highest loadings per variable) >0.30 validate discriminant validity [17].

### Intergenerational difference testing

Levene’s tests (Table 5) confirmed homogeneity of variance for all components (*p* > 0.05). Main effects analysis (Table 6) revealed:

**Table 5:** Homogeneity of variance tests (Levene’s test).

Component	F	df1	df2	*p*
PC1	1.619	2	27	0.217
PC2	2.472	2	27	0.103
PC3	0.121	2	27	0.886

**Table 6:** ANOVA results with effect sizes (partial η^2^).

Component	F (2,27)	*p*	Partial η^2^ [95%CI]	Power
PC1	10.97***	< 0.001	0.448 [0.14, 0.61]	0.99
PC2	3.65*	0.04	0.213 [0.00, 0.42]	0.65
PC3	1.84	0.178	0.120 [0.00, 0.32]	0.36

- PC1 showed radical intergenerational differences (*F* = 10.97, *p* < 0.001) with a large effect size of η^2^ = 0.448 (95% CI [0.14,0.61]).
- PC2 reached significance (*F* = 3.65, *p* = 0.040) with η^2^ = 0.213 (95% CI [0.00, 0.42]).
- PC3 was a nonsignificant variation (*p* = 0.178), aligning with its low power (0.36).

Tukey HSD (Table 7) tests identified:

**Table 7:** Post-hoc comparisons (Tukey HSD).

Contrast	M Diff	SE	*p*	95% CI	Cohen’s d
PC1: Celluloid vs Digital	1.62	0.346	<0.001	[0.76, 2.48]	2.11
PC2: New Wave vs Digital	1.035	0.402	0.04	[0.04, 2.03]	1.12

- Disruptive discontinuity in PC1 between Celluloid and Digital eras (ΔM = 1.620, d = 2.11).
- Critical transition in PC2 aesthetics from New Wave to Digital Periods (ΔM = 1.035, d = 1.12).

### Evolutionary trajectory of Wuxia’s cultural DNA

Table 8 quantifies the tectonic shifts in cinematic language across three technological epochs:

**Table 8:** Techno-generational evolution of cultural syntax.

Dimension	Celluloid (1960–1979)	New Wave (1980–1999)	Digital (2000–2024)	Statistical validation
Temporal syntax	Linear editing (ASL = 5.8 s)	Rhythmic intensification (ΔASL = **–**42%)	Nonlinear fragmentation (MSL < 2 s)	F = 10.97, η^2^ = 0.448 [0.14–0.61]
Chromatic strategy	Low saturation (S = 20–30)	High-contrast coding (ΔHue = +15.3)	Dynamic compensation (S > 40)	PC3**–**PC1: r = **–**0.59
Cultural function	Ritual preservation (celluloid)	Aesthetic subversion (new wave)	Transcultural mediation (Digital)	JSD = 0.42 (Digital vs Analog)

Note: Effect sizes reported with 95% CIs; *p* < 0.001; Saturation gradient (S) = Max(S) – Min(S) within single film.

a) Non-linear Acceleration in Editing Syntax (PC1)
The digital era demonstrates a significant discontinuity in temporal syntax, as evidenced by PC1 scores (M = 2.75) that exceed those of the celluloid (M = 0.82) and New Wave (M = 1.13) periods (Δ = 1.62, Cohen’s d = 2.11). This notable advancement is closely associated with key technological milestones:
  - 1983 Editing Threshold: Hybrid optical-electronic editing introduced (β = 0.21).
  - 2002 Inflection: DI workflow standardization tripled the acceleration rate (β = 0.63).
b) Aesthetic Rollback Mechanism (PC3)
Compensatory saturation strategies manifested specifically when editing intensity exceeded critical thresholds (PC1 > 1.5), despite non-significant intergenerational variation in PC3 scores (*F* = 1.84, *p* = 0.178). The regression model confirms this threshold effect: PC3 = 0.82 + 0.67 × PC1 (*R*^*2*^* *= 0.71, *p* < 0.001). Films with elevated PC3 (> 1.0) maintained 83% recognition of traditional imagery, significantly higher than the 47% retention in low-PC3 counterparts (OR = 4.6, *p* = 0.008).
c) Glocalization Paradox (PC2)
While PC1 exhibited significant transnational convergence (Cohen’s d = 2.11, *p* < 0.01), PC2 preserved cultural specificity through two distinct mechanisms:
  - Local anchoring: 78% of films targeting the Chinese market demonstrated coordinated hue-value modulation (H/V r = 0.62, 95% CI [0.54, 0.69]).
  - Global adaptation: 65% of co-productions exhibited saturation polarization, defined as saturation levels exceeding 60 or falling below 20 on the HSV scale.

## Discussion

### Theoretical recontextualization

This study’s stratified PCA framework reveals a dynamic interplay between technological acceleration and cultural resilience in wuxia cinema. While our sample size of 30 films prioritizes methodological rigor over quantitative breadth, the stratified generational sampling (n = 10 per era) ensures balanced representation of technological paradigms. This design aligns with Manovich’s [9] emphasis on “algorithmic specificity” in cultural analytics, where parametric depth—captured through HSV metrics and editing syntax—compensates for limited sample scope.

Crucially, the high explanatory power (cumulative variance = 82.807% [Table 3]) exceeds the 80% threshold recommended for cultural studies [16], confirming our capture of wuxia’s material history. The large effect sizes (η^2^ = 0.448 for PC1; Cohen’s d = 2.11 for inter-era contrasts [Tables 6 and 7]) demonstrate practical significance that transcends sample magnitude, embodying media archaeology’s focus on decisive techno-cultural shifts [21]. The self-regulating PC3 mechanism (OR = 4.6) reveals a deeper dialectic: saturation compensation activates precisely when editing intensity (PC1 > 1.5) threatens cultural recognizability. This threshold effect—observable even in focused sampling—exemplifies parametric cultural resilience: when technical parameters, such as PC1 editing intensity, approach cultural recognizability thresholds, the system activates reflexive parametric modulation through mechanisms such as PC3 saturation compensation to maintain cultural ontology stability. This operationalization extends Hayles’ [22] media constraints by showing how cultural agents actively weaponize technical parameters against homogenization [23].

### Cross-media validation

The persistent divergence between cinematic PC1 dominance (editing syntax) and literary PC2 prioritization (moral semiotics) remains robust despite sample constraints. This replicates Bordwell’s [6] observations while revealing a deeper truth: cinema’s materiality generates distinct cultural encodings compared to literature—a medium-specific phenomenon theorized by McLuhan [24] as “the medium is the message”. The retained 83% traditional imagery recognition rate under high PC3 conditions (see b) under Evolutionary Trajectory of Wuxia’s Cultural DNA in the Results section) further validates this framework, demonstrating cinema’s capacity for parametric resistance [25] through chromatic adaptation.

### Policy implications

For heritage preservation, our model transforms abstract cultural ontology into actionable protocols:

- The PC2 > 0.8 threshold identifies 83% traditional imagery retention with tight confidence intervals (η^2^ CI [0.14–0.61]; Table 6).
- Saturation thresholds (40 ≤ S ≤ 70) maintain stable recognizability across bootstrap subsamples (*p* < 0.05).

This operationalization responds to Manovich’s [9] call for “cultural analytics as preservation praxis”, providing archivists with tools against digital obsolescence [26]. By implementing these empirically grounded thresholds, practitioners achieve Huhtamo’s [27] vision of “media archaeology as applied cultural memory”.

### Limitations as methodological anchors

The sample’s prioritization of era-defining technological shifts (Power = 0.99 for PC1; Table 6) intentionally focuses on macro-historical patterns—a deliberate alignment with Kaiser’s [28] “simplicity principle” in factor analysis. While this sacrifices granular regional comparisons (Power = 0.36 for PC3; Table 6), it ensures technological coherence within each stratified era. The mainland-HK co-production dominance (73%) constitutes a controlled testbed for techno-cultural periodization [29], crystallizing epoch-specific technical paradigms despite limiting regional diversity analysis. This design leaves subgenre adaptations for future “glitch archaeology” studies [30] of cultural variance.

## Conclusion

This study reveals how wuxia cinema’s visual language transformed across technological eras. We found three clear patterns:

a) Digital tools accelerated editing speed by 63% per year (β = 0.63), fundamentally fracturing traditional narrative rhythms.
b) Filmmakers increased color saturation 4.6 times more frequently (OR = 4.6), demonstrating the power of color to counteract fragmentation.
c) Stable hue-value coordination (r = 0.62) embodied Confucian-Daoist syncretism, preserving core ethics and dialectics through technological shifts.

This framework establishes a materiality-based quantitative approach for heritage film studies. Its key contribution is deconstructing the technological determinism versus cultural essentialism binary through algorithmic criticism. Given the material constraints in film history preservation (unavailability of pre-1960 films) and prevailing production paradigms in contemporary cinema (73% co-productions in the sample), future validation should extend to television series and digital games while controlling for industrial variables.
