# Supplementary material for: Cultural anchoring in cinematic digitalization: Stratified PCA reveals chromatic adaptation regimes in wuxia cinema evolution
Source: PLoS One. 2025 Oct 7;20(10):e0334061. doi: 10.1371/journal.pone.0334061 (PMC12503260; doi:10.1371/journal.pone.0334061)
Supplement: Attachment — Submitted filename: Editor_Comments.docx [file pone.0334061.s001.docx]

## Dear Dr Zhao,

## Your above referenced manuscript, entitled "Cultural Anchoring in Cinematic Digitalization: Stratified PCA Reveals Chromatic Adaptation Regimes in Wuxia Cinema Evolution" requires some revisions before it is ready for reviewing in Plos One. Comments from reviewer 1 are also available. Your submission has been returned to you and is located in your Author Dashboard as a draft, so that you can make the required changes to it and submit it again.

## Overall Assessment:

This study presents a novel and methodologically rigorous approach to analyzing aesthetic and cultural transformations in Chinese wuxia cinema. Its use of computational tools and color-space metrics is particularly innovative. However, the abstract currently prioritizes technical density over accessibility. By slightly simplifying and clarifying key terms and results, the abstract could better communicate the significance of the research to a broader academic audience.

## Recommendations for Revision:

- Clarify and briefly define core concepts and abbreviations.
- Reframe statistical results into more narrative-friendly language.
- Expand slightly on theoretical contributions and implications.
- Prioritize clarity and flow over data density in the abstract format.

This is a highly promising and original contribution. With minor revisions aimed at improving clarity and accessibility, it will be much better positioned to engage readers across disciplinary boundaries.
