# Supplementary material for: Cultural anchoring in cinematic digitalization: Stratified PCA reveals chromatic adaptation regimes in wuxia cinema evolution
Source: PLoS One. 2025 Oct 7;20(10):e0334061. doi: 10.1371/journal.pone.0334061 (PMC12503260; doi:10.1371/journal.pone.0334061)
Supplement: Attachment — Submitted filename: Revised Rebuttal Letter.docx [file pone.0334061.s003.docx]

Revised Rebuttal Letter

Manuscript ID: PONE-D-25-16196

Title: Cultural anchoring in cinematic digitalization: Stratified PCA reveals chromatic adaptation regimes in wuxia cinema evolution

Dear Editor and Reviewers:

The authors would like to express their heartfelt gratitude to the Associate Editor and reviewers for their constructive, supportive, and invaluable feedback. We have thoughtfully considered each comment and made revisions to the manuscript in accordance with the reviewers' suggestions. Below is a summary of these revisions. We truly believe that these changes have greatly enhanced the quality of our manuscript.

Comment 1: Reduce technical density in abstract

We have thoughtfully revised the abstract to improve accessibility while ensuring scientific rigor through three key strategies. First, we transformed isolated statistical parameters into engaging narrative descriptions. For example, in Line 13, we changed "post-2002 (β=0.63/year, ΔM=1.62)" to "cutting rates accelerated by 63% annually (β = 0.63, p < 0.01), disrupting traditional narrative continuity." This approach preserves critical coefficients while streamlining redundant metrics.

Second, we added clear operational definitions for all first-occurrence technical terms. In Line 15, for instance, "PC3 (‘Saturation Compensation’)" was revised to "saturation adaptation (PC3: perceptual compensation mechanism under editing saturation)."

Finally, we implemented a careful parameter filtering process that retains only those coefficients with p<0.05 significance essential for hypothesis testing. These modifications collectively lower technical barriers and enhance conceptual clarity.

Thank you for your understanding as we strive to make our work more accessible!

Comment 2: Expand theoretical implications

We are pleased to share that we have thoughtfully expanded the theoretical framework by incorporating critical insights throughout the manuscript. The abstract now culminates in an important assertion: "61% of digital-era films preserved cultural distinctiveness through strategic HSV manipulation, illustrating cinematic aesthetics’ resilience against technological homogenization." This establishes cultural agency as our cornerstone thesis.

This proposition is theoretically anchored in the introduction with the innovative concept of "chromatic sovereignty," which we define upon its first mention as "the strategic reclamation of color semantics from standardized digital workflows." This reframing allows us to reconsider technological determinism within a Media Archaeology paradigm.

In our discussion, we further validate this framework through cross-cultural analysis, revealing how East Asian filmmakers "actively weaponize technical parameters against homogenization" by employing saturation compensation (PC3) as a self-regulating mechanism when editing intensity (PC1) poses risks to cultural recognizability.

Ultimately, our conclusion articulates a significant disciplinary paradigm shift: "This materiality-based transition—from technological subjugation to chromatic sovereignty—reconfigures media archaeology’s central premise," positioning HSV metrics as valuable tools for deconstructing cultural essentialism. We ensure that all four theoretical innovations come with operational definitions at their point of initiation, thereby maintaining conceptual coherence.

I hope you find this revision helpful!

Comment3：Clarify and briefly define core concepts and abbreviations.

We took great care to ensure that all terminology was clearly defined upon first occurrence, promoting interdisciplinary accessibility. This is particularly important for core technical constructs such as HSV, ASL, and MSL within the data analysis framework. In the Methods section under "HSV color parameter extraction," we explicitly define the HSV color space model as follows: “Hue (the spectral wavelength defining 'color,' such as red or blue), Saturation (the intensity or purity of the color), and Value (its relative lightness or darkness).”

The operational definition of Hue Entropy is presented as: “a measure of color symbol diversity indicating cultural richness,” while Saturation Mean is described as: “reflecting emotional intensity, with high values signaling dramatic tension.” For editing metrics in “Editing rhythm parameter extraction,” Average Shot Length (ASL) is defined as: “a measurement of the global editing pace in action scenes, highlighting directorial control over temporal flow.”

For other key constructs, we operationally define Chromatic Sovereignty in the Results section under "Glocalization Paradox," supported by empirical evidence stating that "61% of digital-era films maintained cultural distinctiveness through strategic HSV manipulation" (Source: Results > Section 4.2). Its theoretical implications are further elaborated in the Discussion section with a statement about "a paradigm shift from technological subjugation to chromatic sovereignty" (Source: Discussion > Paragraph 1).

Additionally, we formally define the original concept Parametric Cultural Resilience within our mechanism analysis in the Discussion section. It states that "when technical parameters (e.g., PC1 editing intensity) approach cultural recognizability thresholds, the system activates reflexive parametric modulation through mechanisms such as PC3 saturation compensation" (Source: Discussion > Paragraph 5).

Comment 4: Improve language fluency

We thoughtfully enhanced linguistic fluency through three key measures:

First, we enriched the text with additional critical transitional sentences. For instance, we revised the "technological black box" discourse to say, "In addressing the epistemic closure within the debate of technological determinism versus cultural essentialism, this study transcends qualitative paradigms by employing a spatio-chromatic triaxial framework," thereby strengthening logical coherence.

Second, we addressed several grammatical inconsistencies by adjusting subject-verb agreement (e.g., changing "employ" to "employs") and refining object components in the Methods section.

Finally, we aimed to improve readability by deconstructing lengthy sentences. For example, we split a 43-word description of the globalization paradox into two concise statements: "Globalization accelerates cinematic homogenization, posing existential threats to cultural identity," and "As Hollywood's technical standards dominate local production—where maintaining aesthetic autonomy becomes crucial—this tension intensifies.".

In addition, to ensure strict language standards, this manuscript was professionally edited by Elsevier Language Services (the certificate is attached below). The editing work focuses on grammar, syntax, and academic style and does not alter the scientific content or interpretation.

Comment 5: Theoretical conclusions underdeveloped

We are pleased to share that we have significantly deepened the theoretical implications of our work through three key enhancements:

Conclusion a: Our finding that "editing speed accelerated by 63% annually (β=0.63)" now explicitly connects to the core thesis of "cultural agency against technological homogenization" (Abstract). This demonstrates how quantitative metrics can effectively reframe discussions around technological determinism.

Conclusion b: The observed "4.6-fold increase in saturation compensation (OR=4.6)" directly operationalizes the mechanism of "parametric resistance through chromatic adaptation" (Discussion), while also highlighting its important role in the "strategic reclamation of color semantics" (Introduction).

Conclusion c: We present the "stable hue-value coordination (r=0.62)" as empirical evidence for "cultural distinctiveness through HSV manipulation" (Abstract). This materializes our concept of a "materiality-based transition from technological subjugation to chromatic sovereignty" (Conclusion).

For clarity, all theoretical terms carry explicit definitions derived from their original contexts:

"Chromatic sovereignty" = “strategic reclamation of color semantics from standardized workflows” (Introduction). "Parametric resistance" = “weaponizing technical parameters against homogenization” (Discussion). "Materiality-based transition" = “reconfiguring media archaeology's central premise” (Conclusion).

Comment 6: Ambiguous statistical reporting

We have made adjustments for clarity to ensure consistency with the revised results:

Temporal discontinuity: “In the digital age, we observe a PC1 discontinuity of ΔM=1.62 compared to the film age (d=2.11), with an acceleration rate of β=0.63/year after 2002” (Table 8).

PCA variance: “PC3 accounts for 16.265% of the unique variance (Table 3) and is activated at a threshold where PC1>1.5” (Results).

Effect size: “The η² value of 0.448 for PC1 surpasses the threshold considered indicative of large effects in cultural studies (η²>0.14 as referenced in [16])” (Table 6 Note).

Comment 7: Clarify data restrictions

The 30 video samples analyzed in this study were legally obtained from licensed streaming platforms in China (Tencent Video, iQiyi, and Youku) and were publicly accessible within Mainland China. Due to copyright restrictions under Chinese law and the Mandarin language content of these films, the original video files cannot be redistributed. However, to ensure transparency and facilitate reproducibility, we have publicly shared detailed access instructions for the martial arts films used and the complete original dataset in a third-party public repository. This includes all extracted HSV parameters (H/S/V values of key frames), editing rhythm metrics (ASL, MSL), and their normalized results. These resources are available under an MIT license at:

<https://github.com/KelsiLiu/Accessible-video-links-and-original-datasets-of-30-wuxia-films.git>

The core analytical findings of this study are robustly validated in Tables 1, 2, 4, and 8. To ensure the complete reproducibility of our analyses, the original, open-source code used for both HSV processing and editing rhythm analysis (ASL/MSL calculation) is freely available in the following repositories under MIT licenses:

HSV parameter extraction: <https://github.com/KelsiLiu/film-HSV.git>

ASL/MSL calculation: <https://github.com/KelsiLiu/film-ASL-MSL.git>

Comment 8: Reference Standardization

We have taken the opportunity to correct any incorrect references, add DOIs, and standardize reference formats for clarity. For instance, the reference for Teo, S. (2015). Chinese martial arts cinema: The wuxia tradition. Edinburgh University Press has been revised to:1. Teo S. Chinese martial arts cinema: The wuxia tradition. Edinburgh: Edinburgh University Press; 2015. https://doi.org/10.3366/edinburgh/9780748632855.001.0001.

Thank you for your understanding as we strive to enhance the quality of our references!
